# ‘Comment J’ai Vaincu La Douler Et L’Inflammation Chronique Par L’alimentation’ by J Lagancé

**Published:** 2012-10-11

**Authors:** Carolina Ciacci

**Affiliations:** Università degli studi di Salerno (cciacci@unisa.it)

Jean Seignalet (1936–2003) worked as a doctor and professor in the Montpellier Hospital (France). Known as a pioneer in renal transplantation, he is also the author of a theory, which states that most autoimmune diseases are related to the modern diet. Dr. Seignalet believed that any *new* food, thus any food added to the archaic diet during the thousands of years of the mankind evolution, may induce immunostimulation and disease because of proteins and other nutrients arenot recognized as self-antigens. Inspired by the scientists Edward Bach (1886–1936), Paul Carton (1875–1947) and Catherine Kousmine (1904–1992), the scientific background of his theory relies on knowledge decades old and mostly unconfirmed by modern research standards. Briefly, he believed that modifications in agriculture over time had generated cereals with toxic proteins. Also, he believed that the modification of proteins of meats and vegetables, which occurs by cooking them at high temperatures as we do in the modern era, had not been accompanied by a similar adjustment of the human digestive system to many food-derived antigens. Thus, the proteins we eat may not be completely digested, causing the intestinal microflora change from fermentative (normal) to putrefactive (abnormal). The products of putrefaction are toxic, and once absorbed unbalance the bodily systems. According to Dr. Seignalet’s theory, these toxins can cause disease processes through many different mechanisms: the elimination of the excess of toxins (acne, aphtosis, irritable bowel disease, allergic congiuntivitis, Crohn’s disease), the accumulation of this excess of toxins (angina pectoris, diabetes, hypercholesterolemia, Parkinson’s disease, hemicranias, osteoporosis) and/or the stimulation of the immune system (arthritis, spondiloarthritis, scleroderma, lupus etc.).

His theories, however, thirty years ago did not engender the favor of the scientific community who called for empirical evidence of such an antigenic response to food. Data from ample patient series on the successful treatment of his diet in those with immunological disease remained unpublished in peer-reviewed journals due to the lack of controls. For this reason in 1998 he published “L’Alimentation ou la Troisième Médecine” (Alimentation, the third medicine) a book in which he exposed the basis of such a diet, its healing/preventive capability and also reported his data of the efficacy of the diet.

More recently, Jacqueline Lagacé a Canadian microbiologist, after successfully curing her own bodily pain due to osteoarthritis with the Seignalet hypotoxic diet, published a book summarizing the principles of the diet. In the book she also described her search for scientific evidence supporting the Seignalet’s recommendations through investigating the most up-to-date published papers.

The book was originally written in French and titled ‘Comment J’ai vaincu la douler et l’inflammation chronique par l’alimentation’ and published in Italy with the title ‘L’alimentazione anti dolore’ (Sperling & Kupfer Ed.). Briefly, Dr. Lagacè summarizes and sustains, with scientific references, Dr. Seignalet’s archaic, hypotoxic diet designed to avoid the ingestion of toxic/ immunogenic food. Its basic principles are:

- Exclusion of cereals, with exception of rice, and of dairy and dairy products. These beliefs are predicated on the knowledge that modern wheat has 21–23 chromosomes, whereas “ancestral” wheat had 7. Rice is allowed because it was not modified in that measure.
- Preferential consumption of raw products (at least 70% of the food should be eaten raw). In fact, cooking food with a temperature over 200ºC forms many mutagens; Maillard molecules and isomers are also created that turn into toxins once inside our bodies.
- Use of virgin oils, obtained by cold pressure (olive oil, walnut, soy and canola oil)
- Priority to biologic products.
- Addition of probiotics.

The reading of this book has been challenging, as a scientist, for me for many reasons.

The book begins discussing the details of Dr. Lagacè’s articular inabilities and the progressive remission of her bodily pain during the hypotoxic diet. Dr. Lagacè, a researcher who also published some scientific papers, well knows that personal, anecdotal experiences, although true and convincing, are still not considered a scientific proof of a theory. However, the personal experience is indeed appealing for an average reader, probably suffering from a chronic disease, adding credibility to the author and her writings.

The original Seignalet classification of disease according to the elimination, accumulation and immune response to toxins appears to be unsupported by any modern scientific evidence.

However, the major weakness of Dr. Lagancé’s whole work, is in my opinion, the selection of the scientific evidences supporting Dr.Seignalet’s theory. It seems that only data supporting the diet are cited, not always in the appropriate manner; the rest is simply ignored. For example, take the case of one of the most well researched diseases, rheumatoid arthritis, which is often cited together with other articular disease such as artrosis and spondyloartritis. Dr. Lagacè reports the numbers and percentages of those with rheumatoid arthritis, from the original Seignalet series along with several different small clinical trials, that were successfully cured supporting the hypothesis of a relation between food and arthritis. However, one of these studies (Skoldstam et al, Rheumatol int 2003) showed the positive effects of the Mediterranean diet (by definition quite rich in cereals and dairy products) in ameliorating physical performances of patients with arthritis. Moreover, the modern scientific literature offers a valuable contribution about diet and arthritis that was, in the book completely ignored. In fact, in 2009 all available data on rheumatoid arthritis and diet interventions were compiled into an accurate metanalysis, which failed to demonstrate any effect of varying diets on articular symptoms. The authors of this analysis, which encompasses 15 different studies for a total of more than 800 patients, concluded that ‘*The effects of dietary manipulation, including vegetarian, Mediterranean, elemental and elimination diets, on rheumatoid arthritis are still uncertain due to the included studies being small, single trials with moderate to high risk of bias. Higher drop-out rates and weight loss in the groups with dietary manipulation indicate that potential adverse effects should not be ignored* ‘. (Cochrane Database Syst Rev. 2009 Jan 21;(1) Dietary interventions for rheumatoid arthritis. Hagen KB, By fuglien MG, Falzon L, Olsen SU, Smedslund G.). The first, French edition of dr. Lagacé book was edited in 2011. Should this metanalysis been cited along with the others which sustained the miraculous effect of the Seignalet diet? I believe that the question: “can a certain diet can be of help in rheumatoid arthritis?” should be addressed by appropriate studies, which the Cochrane review shows, are still lacking.

The references cited by dr. Lagacè, are quite often hardly related to the influence of certain foods in relation to immuno-mediated pathologies. Instead the references refer to pathogenic mechanisms that ‘*might*’ also be genetic, induced by other environmental agent and also antecedent to food ingestion. This is because there are no relevant scientific investigations specifically designed to address this point.

For example, the author cites celiac disease as proof of inability of modern humanity to digest cereals. Indeed, celiac disease has a clear, scientifically proved relation to gluten, which is contained in some proteins from wheat and other cereals. Celiac disease has a genetic basis, as it is necessary that the class II HLA DQ2 DQ8 genes be present to develop the disease. About 30% of people in Western world bear these predisposing genes but only 1% percent of population is affected with celiac disease. Should the remaining 99% of people (70% of them not even at genetic risk for celiac disease) eat a gluten free diet to prevent immuno-mediated diseases? Does the existence of celiac disease help sustain the assertion that the archaic diet can be used to prevent immune-mediated disease? Does it supply a scientifically sound basis?

Dr. Seignalet and Dr. Lagancé claim that IDDM is also related to modern diet and can be prevented/helped by a hypotoxic diet. It has been demonstrated that in IDDM, apart from the genetic background, there is an increased intestinal permeability that allows macromolecules to penetrate the intestinal barrier and cause inflammation. No evidence at the moment is present that this is certainly related to modern cereals. Is any particular food the cause of the appearance of IDDM in genetically predisposed patients? When Seignalet hypothesized the link between modern cereals and intestinal inflammation in IDDM, no published scientific evidence was available to support her claim. Still now, no data demonstrate that any specific diet might prevent or control IDDM besides rigorous glucose monitoring.

Dairy products in the ancient diet were not available and Dr. Seignalet correctly states – as Dr. Lagancé reports - that the protein? content of cow milk is more than 10 times greater than that of human milk. This disparity in exposure to the amount of protein in the early stages of life is thought to be dangerous for the human health, causing major chronic diseases like IDDM, cancer, autism and neurological impairment. To this purpose Dr. Lagancè reports data, recently confirmed, that prolonged breastfeeding favors a better cognitive development. However, again, the data are not at all indicating that it is the quality or the quantity of proteins of milk that influence cognitive behavior. Research in fact suggests that it is likely the relationship of the newborn with a breastfeeding mother that plays a major role. The role of milk proteins in determining cancer, and cardiovascular disease have been object of research, so far though no clinical trial have been produced with favorable results. Only a small, double blind clinical trial, not cited in the book, had indeed demonstrated that autistic children on a casein-gluten free diet had shown no improvement in their behavior when compared to other children on a gluten-rich diet. This implied that the reported beneficial effects of the gluten-free diet might be related to parental placebo effects related to the diet. (The Gluten-Free, Casein-Free Diet In Autism: Results of A Preliminary Double Blind Clinical Trial. Elder JH, Shankar M, Shuster J, Theriaque D, Burns S, Sherrill L. Journal of Autism and Developmental Disorders, Vol. 36, No. 3, 2006)

The average reader may be convinced of the theories presented in the book about the bad effect of casein and milk in general. In fact, milk lactose intolerance causes gastrointestinal symptoms in many people, falsely supporting the idea that milk is not good for our health. Lactase non-persistence (adult-type hypolactasia) is present in more than half of the human population and is caused by the down-regulation of lactase enzyme activity during childhood. However, in the western world, 95 % of the adult population has sufficient lactase levels to drink fresh milk without symptoms. The ability for humans to tolerate dairy is not only the result of a genetic mutation, developed over a about 9000 years of milking cattle, but also is proof of a constant adaptation of the human species to the changing environment. Therefore, milk is certainly one of the nutrients that favors our health and in the long period supported the evolution of mankind.

Another point of discussion in Dr. Langancé’s book revolves around the failure to articulate that body weight loss may be important in reducing the pain of articular disease. Any dietetic intervention that excludes cereals, milk and other dairy products, and increases the intake of vegetables will likely cause weight loss. I believe that the evaluation of body weight before and after diet should have been an object of analysis.

In conclusion, the hypothesis that certain foods, or, better, certain proteins present in the modern diet are linked to chronic diseases is fascinating and deserves significant scientific attention. But evidence-based medicine must now be the reference for any health issue. Scientific research methods require objectivity and control of findings through the randomization of subjects participating in the study. The Seignalet diet was not studied in controlled clinical trials. Therefore the Seignalet theory leaves much to be demonstrated. Dr. Lagancè in her investigations also fails to bring to light this point. Nutritionists teach us that any intervention on the diet with any special diet - in particular if not supported by strong evidence-may cause an unbalance of food composition.

Finally, to nowadays there are no data supporting the theory that raw meat, fish and poultry are safe for human. Raw foods may even be dangerous, for example exposing the population to helminths and bacteria.

In conclusion, the scientific world that at that time ignored dr. Seignalet unproven theories should now continue to fight and compel those who prescribe elimination diets to prove their efficacy first. Divulgating poorly-controlled, non-evidence-based medical information increases confusion and generates doubts and unnecessary health interventions. Psychological costs of such a diet have not been taken into consideration but the celiac disease experience tells us that any restrictive diet, even when necessary, causes reductions in quality of life.

